# Maternal Stress Reduces the Susceptibility of Root-Knot Nematodes to *Pasteuria Penetrans*

**DOI:** 10.21307/jofnem-2019-040

**Published:** 2019-07-29

**Authors:** Chang Liu, Pingsheng Ji, Patricia Timper

**Affiliations:** 1 Department of Plant Pathology, University of Georgia, Tifton, GA, 31793; 2 Entomology and Nematology Department, 1881 Natural Area Dr, Gainesville, FL 32611; 3 USDA ARS, P.O. Box 748, Tifton, GA, 31793

**Keywords:** Biological control, Crowding, Host-parasite interactions, *Meloidogyne arenaria*, Transgenerational immune priming

## Abstract

*Pasteuria penetrans* is an obligate parasite of root-knot nematodes (*Meloidogyne* spp.). Endospores of *P. penetrans* attach to the cuticle of second-stage juveniles (J2) and complete their life cycle within the nematode female body. Infected females will be filled with spores and will be sterilized. Studies with *Daphnia magna* and its parasite *Pasteuria ramosa* showed that a poor maternal environment can lead to offspring resistant to *P. ramosa*. Therefore, we hypothesized that *Meloidogyne arenaria* females raised under a stressed environment would produce offspring that were more resistant to *P. penetrans*. Females were exposed to a stressed environment created by crowding and low-food supply, or a non-stressed environment and their offspring evaluated for endospore attachment and infection by *P. penetrans*. No difference in spore attachment was observed between the two treatments. However, infection rate of *P. penetrans* in the stressed treatment was significantly lower than that in the non-stressed treatment (8 vs 18%). Mothers raised under stressed conditions appeared to produce more resistant offspring than did mothers raised under favorable conditions. Under stressful conditions, *M. arenaria* mothers may provide their progeny with enhanced survival traits. In the field, when nematode populations are not managed, they often reach the carrying capacity of their host plant by the end of the season. This study suggests that the next generation of inoculum may be more resistant to infection by *P. penetrans*.

Phenotype is a complex interaction between genotype and environment (Bernardo, 1996; Mousseau and Fox, 1998). Environmental influences on the phenotype can occur indirectly through the mother based on cues she receives from the environment. The mother then alters the phenotype of her offspring to prepare them for the environment they will experience. One of the most well-studied maternal effects is the transfer of disease resistance from vertebrate mothers to their offspring. Vertebrates mothers can pass on immune factors, such as antibodies, to their progeny through colostrum, milk, yolk eggs, etc. (Brambell, 1970; Grindstaff et al., 2003). Invertebrates lack the adaptive immune response of vertebrates; nevertheless, maternal effects on innate immunity have also been observed in several invertebrates in response to exposure to parasites or immunostimulants as reviewed in Pigeault et al. (2016). This response is termed transgenerational immune priming and can lead to a wide spectrum of resistance specificity, from general to highly pathogen specific (Moret and Schmid-Hempel, 2001; Little et al., 2003; Dubuffet et al., 2015; Dhinaut et al., 2018).

Exposure to substandard environmental conditions can also influence an organism’s susceptibility to disease. When female water fleas, *Daphnia magna,* were exposed to low-food levels or high temperatures, their offspring were more resistant to the pathogen *Pasteuria ramosa* than were offspring of females exposed to adequate food levels and moderate temperatures (Mitchell and Read, 2005; Garbutt et al., 2014; Garbutt and Little, 2017). Similarly, Boots and Roberts (2012) reported that Indian meal moth (*Plodia interpunctella*) offspring from mothers provided low quality food were more resistant to an insect virus (PiGV). Moreover, these offspring also had higher phenoloxidase activity, an indication of general immune response, than offspring from mothers given high quality food. These observations may be part of a general phenomenon by which mothers optimize their reproductive allocation strategy. Mothers experiencing harsh environments produce fewer eggs and invest more in offspring which are larger in size and have greater survivorship when faced with starvation and disease (Gliwicz and Guisande, 1992; Mousseau and Fox, 1998; Garbutt and Little, 2017). Maternal effect can vary between different host genotypes, indicating there are genotype by maternal environment interactions (Stjernman and Little 2011). When different *D. magna* genotypes were raised under low-food conditions, their offspring showed varying levels of resistance to parasites.

*Pasteuria penetrans* is an obligate bacterial pathogen of root-knot nematodes, *Meloidogyne* spp. The infection process of *P. penetrans* has multiple steps during which the nematode could resist infection. The first step is attachment of endospores to the cuticle of the second-stage juvenile (J2). Individuals within a root-knot nematode population have been shown to vary in their susceptibility to attachment by endospores of *P. penetrans* (Trudgill et al., 2000; Timper, 2009). Moreover, Tzortzakakis et al. (1996) demonstrated that a population of *Meloidogyne javanica* developed resistance to endospore attachment when repeatedly challenged with an isolate of the bacterium. The second step of infection is penetration of nematode cuticle by a germ tube (Imbriani and Mankau, 1977; Sayre and Wergin, 1977). Although only 20 to 30% of endospores that attach to the cuticle infect the nematode (Stirling, 1984; Rao et al., 1997), it is assumed that spores that do not infect are not viable. However, it is also possible that the nematode mounts a defense against spore penetration of the cuticle. Pujol et al. (2008) documented increased expression of antimicrobial peptides in the epidermis of *Caenorhabidits elegans* during infection by the pathogenic fungus *Drechmeria coniospora*. Overexpression of the antimicrobial genes resulted in greater resistance to *D. coniospora*. Following successful penetration, *P. penetrans* forms either microcolonies or filamentous structures (rhizoids) that extend into the nematode pseudocoelom (Davies et al., 2011). During these early stages of infection, the nematode may produce antimicrobial peptides to prevent proliferation (Pillai et al., 2003). In the later stages of infection, sporogenesis occurs and mature endospores are formed within the body of the female nematode. In a recent study, prior exposure of *Meloidogyne arenaria* J2 to root exudates from both host and non-host plants reduced attachment of *P. penetrans* compared to J2 that had no prior exposure to root exudates (Liu et al., 2017). This was the first evidence that cues from the environment could alter the susceptibility of root-knot nematodes to pathogens. The objective of this study was to investigate the influence of maternal stress in *M. arenaria* on the susceptibility of their offspring to parasitism by *P. penetrans*. Because host genotype can influence the outcome of maternal effects on host immunity (Hall and Ebert, 2012), two single egg mass lines (SEM) of *M. arenaria* were evaluated for their response to maternal stress.

## Materials and methods

### Rearing females under stressed and non-stressed conditions

Females of *M. arenaria* were exposed to stress from crowding which likely also resulted in low nutritional levels. The experimental design was a 2 × 2 factorial with two treatments (stressed and non-stressed) and two single egg mass (SEM) lines of *M. arenaria* (SEM 23 and 40). The two SEM lines were obtained from a field population in Tifton, GA, and maintained on eggplant (cv. Black Beauty) in a greenhouse. To initiate the experiment, four-week-old eggplant seedlings were transferred to 10 × 10 cm pots containing 700 cm^3^ of a pasteurized loamy-sand soil one week before nematode inoculation. Nematode inoculum was obtained from four-month-old nematode cultures by cutting the roots from the plant, washing them in tap water, and placing the roots in a mist chamber for egg hatch. Newly hatched J2 were collected every two days. For the stressed treatment, 5,000 J2 were inoculated into each pot and the lower two leaves were pruned to reduce root growth (Snyder et al., 2006). For the non-stressed treatment, 1,000 J2 were inoculated into each pot and the leaves were not pruned. There were five replicates per treatment combination. Two months after inoculation, the presence of root-knot nematode males was used as an indicator of crowding/low nutrition in the pots. Males of parthenogenic *Meloidogyne* spp., such as *M. arenaria*, undergo sex reversal induced by unfavorable environmental conditions (Triantaphyllou, 1973) such as crowding, loss of host foliage, or host resistance (Davide and Triantaphyllou, 1967, 1968; Moura et al., 1993). To determine the number of males, five soil cores were removed from each pot with a small sampling probe (15 mm dia.). The nematodes were extracted from 100 cm^3^ of soil by the centrifugal sugar floatation method (Jenkins, 1964) and observed under 40× magnification to determine the number of males.

### *Pasteuria penetrans* culture

A single spore line (SS 17) of *P. penetrans* was obtained from University of Florida (Joseph et al., 2017). Spores of *P. penetrans* were produced by inoculating 200 J2s attached with an average of 2 to 5 spores/J2 onto four-week-old eggplant seedlings. The plants were grown in a greenhouse for four months before root harvest. Harvested roots were washed with running water and placed in a beaker containing 100 ml of 10 g Lallzyme EX-V (Lallemand, Montreal, Canada) to digest root tissue for 1 d on a shaker (100 rpm). Fully opaque infected females were freed from roots and handpicked into a glass petri dish containing deionized water (dH_2_0). Females were crushed with a dissecting needle to free endospores into dH_2_O. The spore solution was vacuum filtered (8–12 μm) to remove the female cuticle. Spore concentration was determined under 1,000× magnification on a hemocytometer.

### Evaluating offspring for resistance to *P. penetrans*

To obtain offspring of *M. arenaria*, roots from each pot were cut off, washed, and placed separately in a mist chamber for eggs to hatch. Hatched J2 were collected 3 to 4 d later. A spore attachment bioassay was carried out with J2 from each pot. A 1 ml suspension containing 10^5^ spores was added to a small (50 × 15 mm) glass Petri dish along with 500 J2 and 4 ml of 1× phosphate buffered saline (PBS, pH = 7.4). Dishes were placed on a horizontal shaker at 100 rpm for 6 hr at room temperature (24–26°C). The number of spores attached to 25 randomly selected J2 was determined under 400× magnification. These spore-incubated J2 were then immediately inoculated into new pots containing a single four-week-old eggplant seedling to determine the rate of infection by *P. penetrans*. One month after inoculation, eggplant roots were cut off and washed with tap water. Roots were treated with Lallzyme EX-V as described above. After the root tissue was softened, 30 females per pot were randomly selected and handpicked into glass dishes containing water with the aid of a dissecting microscope. Spore-infected females were fully opaque, while non-infected females were semi-transparent. The number of infected vs non-infected females were recorded. The experiment was conducted twice.

### Effect of stress on offspring number and size

A separate experiment was conducted to determine whether crowding/low-food level of the mother affected the size and number of eggs produced. The experiment was a factorial design with two maternal environments (stressed and non-stressed) and two SEM lines (23 and 40). The methods of subjecting female *M. arenaria* to either stressed or non-stressed conditions were the same as described above. There were four replicate pots per treatment combination. The presence of males was used as an indicator of stress and two months after inoculation, egg masses were handpicked randomly from 10 females per pot. The egg masses were placed individually in a 1.5-ml microcentrifuge tube containing 1 ml of 1.65% NaOCl and agitated on a vortex mixer (Model K-500-J, Scientific Industries, Inc.) for 30 sec to separate the eggs from the mass. The number of eggs per mass was counted at 40× and the volume (based on a prolate spheroid) of five eggs from six egg masses (*n* = 30 per replicate) was determined at 400× using an optical micrometer. The experiment was conducted one time.

### Statistical analysis

For the spore attachment assay and the offspring size and number experiments, data were analyzed using the standard least squares option (analysis of variance) in JMP Pro (v.13). For the spore attachment assay, the mean spores per J2 for 25 individuals were used as the data points. The model included SEM line, Treatment, Trial, and all two-and three-way interactions. LS means Student’s *t*-test was used to conduct pairwise comparison of model effects. For the offspring size and number experiment, the mean number of eggs per female and the mean egg volume per pot were used as data points (*n* = 4). The model included SEM line, Treatment, and SEM × Treatment interaction. For female infection rate, binary variables were used, where infected females were represented by 1 and non-infected females were represented by 0. Data were analyzed using the binomial distribution option in JMP. The model included SEM line, Treatment, Trial, and their interactions.

## Results

**Figure 1:**
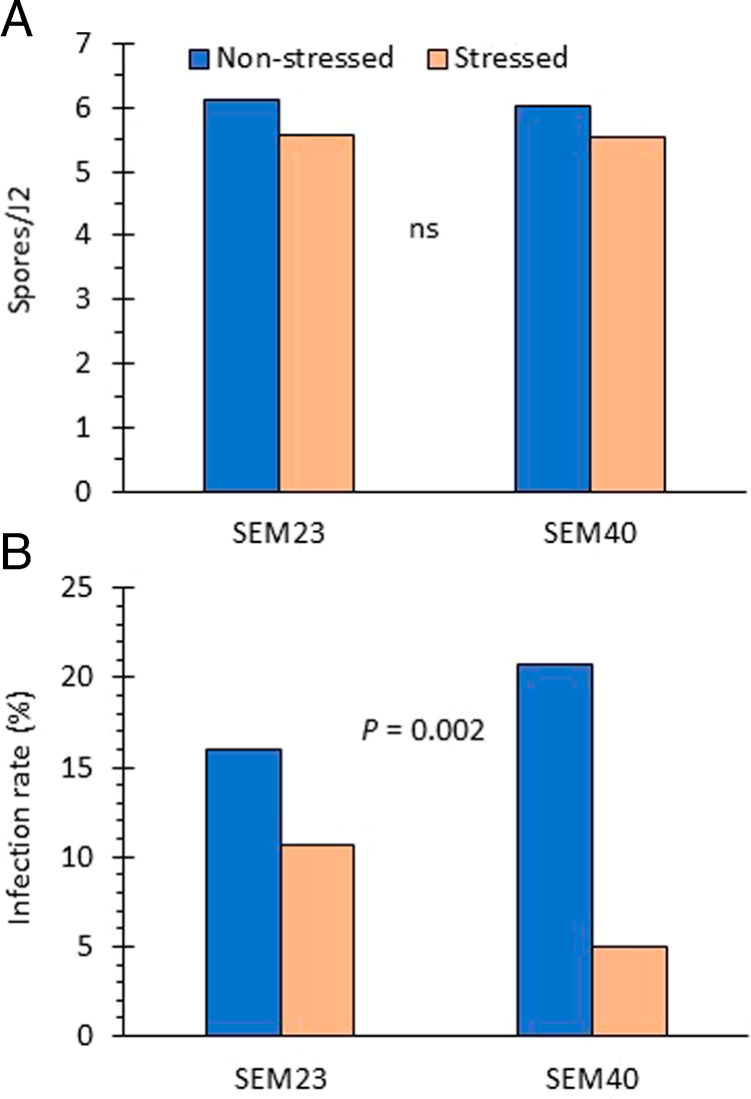
Effect of maternal stress from crowding on the susceptibility of *Meloidogyne arenaria* offspring to (A) endospore attachment; and (B) infection by *Pasteuria penetrans*. Two single egg mass (SEM) lines of *M. arenaria* were evaluated; there was no Stress × SEM interaction. Bars are the mean of two trials and five replications (*n* = 10).

Males were not found in any pots receiving an initial inoculum of 1,000 J2 per pot (non-stressed treatment). In pots receiving an initial inoculum of 5,000 J2 (stressed treatment), the mean number of males per 100 cm^3^ soil was 3.6 in Trial 1 and 3.4 in Trial 2. There was no effect of maternal stress on spore attachment to the offspring (Fig. 1) and this was consistent among trials and SEM lines (no SEM × Treatment or Trial × Treatment interaction). Mean spore attachment in the stressed vs non-stressed treatment was 5.6 and 6.1 spores/J2, respectively. Following inoculation of these J2 onto eggplant, however, fewer (*p* = 0.0016) individuals from mothers reared under stress conditions became infected by *P. penetrans* than from mothers reared without stress (Fig. 1). This difference was consistent between the SEM lines and trials. In the stressed treatment, 7.8% of females were infected with *P. penetrans*, while in the non-stress treatment 18% were infected. None of the infected females produced eggs in this experiment.

In the experiment to evaluate the effect of maternal stress on offspring number and size, the mean number of males per 100 cm^3^ of soil was 1.8 and 2.0 for SEM23 and SEM40, respectively, for the stress treatment. There were no males found in the non-stress treatment. The number of eggs per egg mass did not differ (*p* = 0.18) between the stress and the non-stress treatments (429 vs 462) and this result was consistent among SEM lines. The volume of the eggs, measured as a prolate spheroid (pl), also did not differ (*p* = 0.65) between stress and non-stress treatments (82.2 vs 82.9) and this was consistent among SEM lines.

## Discussion

In this study, we showed that *M. arenaria* females raised under stressful conditions (crowding/low-food levels), but not previously exposed to *P. penetrans*, produced offspring that seemed to be more resistant to infection by the bacterium compared to offspring of females raised without stress and this result was consistent among the two SEM lines. These results are suggestive of transgenerational immune priming, which has not been demonstrated in a nematode before. In *C. elegans*, maternal effects have been reported but these do not involve the immune system. For example, *C. elegans* exposed to pathogenic bacteria produce offspring that enter the dauer stage to avoid infection (Palominos et al., 2017) and adults exposed to osmotic stress produce offspring that are resistant to these conditions (Frazier and Roth, 2009). The mechanism by which *M. arenaria* females enhance resistance to *P. penetrans* in their offspring is unknown but appears to occur after endospore attachment. Offspring of females under stress did not differ in the number of attached endospores compared to offspring of females not under stress. Similarly, host food levels, crowding, and temperature did not affect attachment of *P. ramosa* endospores to *D. magna* (Duneau et al., 2011).

We observed a very low rate of infection by attached *P. penetrans* endospores, even in the non-stress treatment where only 18% of females were infected despite an average of 6.1 spores/J2. Other studies have also reported low rates of infection by *P. penetrans* with only 20 to 30% of attached endospores successfully initiating infections (Sayre and Wergin, 1977; Stirling, 1984; Rao et al., 1997). Yet, Stirling (1984) achieved 90% infection of females with 5 spores/J2, whereas Rao et al. (1997) achieved 40% infection with 3 to 5 spores/J2. It is unclear why only 18% of females in our study became infected. Juveniles encumbered by seven or more endospores have limited mobility and penetrate roots at a lower rate than juveniles with fewer spores (Davies et al., 1991). In our study, 41% of the J2 had seven or more attached spores and few of these J2 may have entered the roots to become infected. There may also be variation among populations in host defense against infection or pathogen virulence. We used a single spore line of *P. penetrans* compatible in attachment to SEM 23 and 40; however, we did not asses its level of virulence to *M. arenaria*.

Our results are in alignment with other studies demonstrating that invertebrates raised under stress conditions produce offspring that are resistant to disease (Mitchell and Read, 2005; Boots and Roberts, 2012; Garbutt et al., 2014; Garbutt and Little, 2017). When the maternal environment is poor, mothers may invest more in each offspring (Beckerman et al., 2006; Boots and Roberts, 2012). Female *D. magna* females raised under stressed conditions produced fewer eggs that were larger size (Gliwicz and Guisande, 1992). Similarly, when *C. elegans* females were reared in a low-food environment, they produced larger eggs and offspring that were more fit in a food scarce environment (Harvey and Orbidans, 2011). Such trade-offs in offspring size and number may be a strategy for mothers to provide their offspring for greater survival (Gliwicz and Guisande, 1992; Rossiter, 1996), including by boosting innate immunity. In *D. magna*, offspring are larger when mothers are exposed to food or heat stress, and large size is associated with resistance to *P. ramosa* (Garbutt et al., 2014; Garbutt and Little, 2017). In our study, we did not observe a difference in either egg number or size between *M. arenaria* females reared in stressed and non-stressed environments. Our method for imposing stress on the nematodes was imprecise and our only measure of stress was the presence of male *M. arenaria*. It is possible that in the experiment to determine egg size and number, the level of maternal stress was insufficient to affect the offspring size or resistance to *P. penetrans*. Unfortunately, we did not measure offspring size and resistance to *P. penetrans* in the same experiment.

Considerable research on innate immunity in nematodes has been conducted with *C. elegans*. The immune response in this nematode is inducible and begins with pathogen recognition followed by activation of one or more signal transduction pathways leading to the production of effector molecules to destroy the invader (Millet and Ewbank, 2004; Gravato-Nobre and Hodgkin, 2005). Many of these signaling pathways are also shared with responses to stress such as wounding, heat, hyperosmotic conditions, and heavy metal exposure (Gravato-Nobre and Hodgkin, 2005; Irazoqui et al., 2010; Ermolaeva and Schumacher, 2014). Nutritional deficiencies may also influence innate immunity in nematodes. Polyunsaturated fatty acids (PUFAs) are essential for many cellular functions, including immunity. Using *C. elegans* mutants defective in the biosynthesis of two PUFAs (gamma-linolenic acid and stearidonic acid), Nandakumar and Tan (2008) showed that they were essential for a functioning p38 MAP kinase immune pathway and resistance to pathogen infection.

Dietary deficiency in PUFAs has been shown to play a role in maternal effects on immunity in *D. magna* (Schlotz et al., 2013). A diet rich in PUFAs increased resistance to *P. ramosa* compared to a diet deficient in PUFAs for the generation consuming the different diets. The reverse was true, however, for the second generation where offspring from mothers provided a diet rich in PUFAs produced more eggs but were six-fold more susceptible to the bacterium. Because PUFAs are substrates for hormone-like compounds (eicosanoids) important for both immunity and reproduction, it has been proposed that there is a trade-off between these two functions, particularly when resources are limited (Schlotz et al., 2013, 2016).

We observed lower rates of infection by *P. penetrans* in offspring from *M. arenaria* mothers raised under crowded conditions than for mothers raised without crowding suggestive of transgenerational immune priming. Alternatively, it is possible that offspring produced by mothers under stress are less vigorous than offspring produced without maternal stress and the burden of endospores has a greater effect on limiting their mobility, thus leading to fewer infected females in the roots. This latter explanation is unlikely because we found no effect of maternal stress on offspring size; moreover, maternal food stress did not reduce offspring survival, a proxy for vigor, in either *Daphnia* or *Drosophila* (Prasad et al., 2003; Guinnee et al., 2007). Additional research is needed to determine whether immune molecules are elevated in offspring from *M. arenaria* mothers raised under crowded conditions.

Maternal effects on root-knot nematode susceptibility has practical implications for managing these nematodes with *P. penetrans* and, perhaps, other microbial control agents. When the nematodes reach the carry capacity of their host plant, which often happens at the end of the crop season when nematode populations are large, the next generation may be more resistant to infection by *P. penetrans*. These resistant offspring will be the initial inoculum for next spring. Therefore, to maximize the efficacy of *P. penetrans*, root-knot nematodes should be managed with crop rotation, host-plant resistance, and nematicides to keep populations low. Moreover, other stressors such as high temperatures and toxins can induce an immune response in other invertebrates and may do the same in root-knot nematodes (Ermolaeva and Schumacher, 2014; Garbutt et al., 2014).
